# Prevalence, predictors, and patterns of patient reported non-motor outcomes six months after stroke: a prospective cohort study

**DOI:** 10.1016/j.lanepe.2024.101080

**Published:** 2024-10-19

**Authors:** Hatice Ozkan, Gareth Ambler, Gargi Banerjee, John J. Mitchell, Carmen Barbato, Simone Browning, Alex P. Leff, Robert J. Simister, David J. Werring

**Affiliations:** a UCL Queen Square Institute of Neurology, London, UK; b National Hospital for Neurology and Neurosurgery, University College London Hospitals NHS Foundation Trust, Queen Square, London, UK; c Department of Statistical Science, University College London, Gower Street, London, UK; d Department of Targeted Intervention Division of Surgery and Interventional Sciences, University College London, London, UK; e MRC Prion Unit at UCL, Institute of Prion Diseases, London, UK

**Keywords:** Patient reported health, Non-motor outcomes, Quality of life after stroke, Neuropsychological, Sleep, Autonomic, Sensory, Language, Life satisfaction, Memory outcomes

## Abstract

**Background:**

Adverse non-motor outcomes have a major impact on patients and caregivers after stroke, but knowledge of their prevalence, predictors and patterns across multiple health domains remains limited; we therefore aimed to obtain these data in a large observational prospective cohort study.

**Methods:**

We included data from the Stroke Investigation Group in North and Central London (SIGNAL) registry based at the University College London Hospitals (UCLH) Comprehensive Stroke Service which serves a multi-ethnic population of ∼1.6 million people. In adult patients diagnosed with acute stroke due to cerebral ischaemia or intracerebral haemorrhage (ICH) from January 2017 to January 2020 we evaluated non-motor outcomes (anxiety, depression, fatigue, sleep disturbance, social participation, pain, bowel dysfunction, bladder dysfunction, mood problems, communication problems, activities of daily living (ADL), memory and thinking problems) at 6-month follow-up. We evaluated baseline predictors in multivariable logistic regression, and correlations between domains using kappa statistics.

**Findings:**

Follow-up was complete for 3080 of 3338 (92.3%) eligible surviving patients (2534 ischaemic stroke, 547 with ICH; mean age 71.2 years, 1379 (44.8%) female, 1774 (59.3%) white). The most prevalent adverse non-motor outcomes were fatigue 1756 (57%), reduced social participation 1694 (55%), sleep disturbance 1663 (54%), and constipation 1355 (44%). The rates of adverse non-motor outcomes in ⩾ 1, ⩾ 2, ⩾ 3, ⩾ 4, and ⩾ 5 domains were 2310 (75%), 1571 (51%), 1519 (49%), 1232 (40%), and 801 (26%), respectively. Factors associated with adverse non-motor outcomes included stroke due to ICH, stroke severity, previous stroke, or history of cardiovascular disease. We identified moderate correlations between fatigue and sleep disturbance (kappa = 0.72); memory and thinking impairment and reduced ADL (kappa = 0.68); and communication problems and ADL (kappa = 0.70).

**Interpretation:**

Adverse non-motor outcomes are highly prevalent and often multiple at 6-months after stroke: 75% have at least one affected domain; fatigue, sleep disturbance, and reduced social participation each affect over 50% of survivors, and 26% of patients report ≥5 adverse outcomes. Our findings suggest an urgent need to better detect and mitigate these outcomes to improve quality of life after stroke.

**Funding:**

The 10.13039/501100000272National Institute for Health and Care Research (NIHR) UCLH Biomedical Research Centre.

**Research in context**

**Evidence before this study**

We conducted a search on PubMed and Ovid databases (including MEDLINE, Embase, and PsychINFO) for articles reporting non-motor outcomes following stroke, using keywords including “non-motor outcomes,” “ischaemic stroke,” “intracerebral haemorrhage,” or “stroke,” published between January 1, 1978, and December 1, 2023. We included studies assessing non-motor outcomes at 6 months or later after acute ischaemic stroke or intracerebral haemorrhage (ICH), excluding those focusing on transient ischaemic attack (TIA) or subarachnoid haemorrhage (SAH) and. Most previous studies focused on single non-motor domains, often with small cohort sizes, and did not provide details on co-occurrence of domains. They mainly included only patients with ischaemic stroke; only a few examined single non-motor domain outcomes after stroke due to ICH. Nevertheless, the limited previous studies available investigating predictors of adverse outcomes in single non-motor outcome domains identified age, sex, and stroke severity (measured by the National Institute of Health Stroke Severity Score (NIHSS)) but findings were inconsistent; very few data compared non-motor outcomes after ischaemic stroke and ICH.

**Added value of this study**

To the best of our knowledge, ours is the first comprehensive investigation into the prevalence and associated factors of adverse non-motor outcomes across multiple health domains in a large, unselected cohort of patients with acute ischaemic stroke and ICH. We collected data from 3080 patients (2534 ischaemic stroke, 547 ICH) in a large prospective cohort study, with 92.3% follow-up completeness at 6 months. This systematic approach allowed an in-depth examination of lived-experience post-stroke across a wide range of non-motor domains to provide a comprehensive understanding of the prevalence, patterns and predictors of adverse non-motor outcomes. At 6 months post-stroke, the most prevalent adverse non-motor outcomes were fatigue (57%), reduced social participation (55%), sleep disturbance (54%), and constipation (44%). The rates of adverse non-motor outcomes across multiple domains were reported by 75%, 51%, 49%, 40%, and 26% of patients in ≥1, ≥2, ≥3, ≥4, and ≥5 domains, respectively. Sociodemographic and clinical factors associated with multiple adverse non-motor outcomes included ICH, admission NIHSS score >5, previous stroke or TIA history, and cardiovascular disease history. We identified correlations between several non-motor outcomes: fatigue and sleep disturbance (k = 0.72), memory and thinking impairment and reduced Activities of Daily Living (ADL) (k = 0.68), and communication problems and ADL (k = 0.70). These findings show a previously unsuspected high and complex burden of multiple non-motor outcomes after stroke, highlighting a major unmet healthcare need. Our data should prove useful to clinicians, patients and policymakers to inform patient-centred care pathways and a more holistic approach to stroke care in order to detect and mitigate these adverse non-motor outcomes.

**Implications of all the available evidence**

Most individuals surviving 6 months post-diagnosis of acute ischaemic stroke and ICH will experience adverse non-motor outcomes across multiple health domains, yet often lack access to screening, targeted interventions, or support plans. Fatigue, reduced social participation, sleep disturbance, and constipation, are particularly burdensome. Several predictive factors have been identified, including stroke severity; however, stroke due to ICH remains understudied despite the apparent very high risk of adverse non-motor outcomes. Taken together, our findings and previous studies underscore the importance of detecting and addressing adverse non-motor outcomes across multiple-domains, and provide helpful data to guide clinicians, patients, carers and policymakers. Nevertheless, further studies are needed to confirm our findings, develop effective care pathways, and to investigate the longer-term burden of adverse non-motor outcomes beyond 6 months after stroke.

## Introduction

Stroke is the third leading cause of death and a major cause of disability-adjusted per person life-years globally.^1^ Traditionally, disability after stroke is assessed using function or impairment-based measures such as the modified Rankin Scale (mRS) or the National Institutes of Health Stroke Scale (NIHSS).^2^ These measures primarily evaluate motor outcomes, neurological deficits or functional independence.^3^ However, stroke-related brain injury can also affect multiple patient-reported non-motor health domains. These can be grouped into the following domains: neuropsychiatric (i.e., anxiety, depression, fatigue, sleep disturbances, and mood problems); sensory (i.e., pain); autonomic (i.e., constipation and bladder dysfunction); cognitive (i.e., memory and thinking); social (i.e., social participation, social relationships); activities of daily living (ADL); and communication (i.e., aphasia).^3^ Although historically overlooked by clinicians and researchers, non-motor outcomes are increasingly recognised after stroke, with major adverse impacts on both patients and their caregivers^3^ including reduced quality of life, inability to return to work, increased dependency, social isolation, and an association with premature death; indeed, non-motor outcomes may have greater health impact than more obvious motor outcomes,^3^, ^4^, ^5^, ^6^, ^7^, ^8^, ^9^, ^10^, ^11^ and have now become a component of recommended stroke care protocols, which emphasise the need for a comprehensive, patient-centred approach to post-stroke disability assessment and treatment planning.^5^, ^6^, ^7^, ^8^

Despite increasing awareness, there is limited data on the prevalence, predictors, and patterns of non-motor outcomes after stroke. Previous studies have been limited by their focus on single health domains,^3^^,^^5^, ^6^, ^7^, ^8^, ^9^, ^10^, ^11^ primarily in cohorts of patients with ischemic stroke at variable intervals post-stroke.^3^^,^^5^ Nevertheless, in recent years several studies have used patient-reported health outcome measures to investigate multiple non-motor outcome domains and associated factors.^12^^,^^13^ However, previous research on predictors of non-motor stroke outcomes are inconsistent: associations with male or female sex and links to functional disability measured by modified Rankin Scale (mRS) are mixed.^5^, ^6^, ^7^, ^8^, ^9^, ^10^, ^11^, ^12^, ^13^ While National Institute of Health stroke severity scale (NIHSS) has been associated with neuropsychiatric and sensory domains of non-motor outcomes, knowledge of associations with autonomic dysfunction and other non-motor outcomes remains limited.^4^^,^^9^ Therefore, the findings of the previous studies, limits comprehensive understanding of multi-domain non-motor outcomes across ischaemic and haemorrhagic stroke subtypes.

In this study, we therefore aimed to: (1) identify the prevalence of non-motor outcomes across 13 health domains (anxiety, depression, fatigue, sleep disturbance, social participation, pain, bowel dysfunction, bladder dysfunction, mood problems, communication problems, activities of daily living, memory and thinking problems); (2) determine the factors associated with adverse non-motor outcomes in each domain; and (3) describe co-occurrence and correlations between adverse non-motor outcomes.

## Methods

### Study design and participants

Participants were recruited from the Stroke Investigation in North Central London (SIGNAL) registry, a population-based, prospective cohort study established in 2015. SIGNAL included consecutive patients with acute ischaemic stroke or ICH, confirmed by brain imaging, in adults (>18 years old) admitted to the hyperacute stroke unit (HASU) within University College London Hospitals (UCLH) Comprehensive Stroke Service. The service serves an ethnically diverse population of 1.6 million people resident in five North Central London (NCL) boroughs: Barnet; Camden; Enfield; Haringey; and Islington.^14^ All patients with suspected stroke within this population are taken directly to the HASU for specialist assessment, diagnosis, stabilisation and hyperacute care before discharge or transfer to step-down local stroke units. In the present study we included all SIGNAL participants recruited from January 2017 to January 2020 who provided at least two non-motor outcome assessments at 6 month follow up.

Our study team visited the HASU at UCLH and reviewed all electronic health records from all hospital admissions. We confirmed stroke diagnosis using all available neuroimaging and clinical reports. We also verified patients' residential postcodes. At stroke unit discharge, patients and their caregivers were informed that they would have follow-up within NCL stroke services, via telephone, postal mail, or home visits at 6 months.

We excluded patients diagnosed with transient ischaemic attack (TIA) or subarachnoid haemorrhage (SAH) because they have different in short- and longer-term neurological impairments and functional consequences in comparison to patients with stroke.

### Baseline data

We obtained the baseline sociodemographic (age, sex, and ethnic origin), and clinical (stroke type, previous medical history, pre-morbid mRS, admission NIHSS, and discharge mRS) data from electronic health records (Epic, Epic Systems Corporation).

### Outcomes

Clinically trained practitioners routinely administered patient-reported non-motor outcome questionnaires, using pre-specified validated measures across 13 non-motor outcome domains (anxiety, depression, fatigue, sleep disturbance, social participation, pain, bowel dysfunction, bladder dysfunction, mood problems, communication problems, activities of daily living, memory and thinking problems). The outcome measures included: Patient-Reported Outcomes Measurement Information System—29 (PROMIS-29); Stroke Impact Scale—59 (SIS-59); and the Barthel Index Score.^15^, ^16^, ^17^ Definitions of each non-motor outcome evaluate in our study are presented in the Supplementary File.

To maximise follow-up and reduce patient burden we offered patients multiple options to provide non-motor outcome data including local stroke outpatient clinics, telephone interview, face-to-face appointment at their residential address, or via post for the small proportion of individuals who required proxy assistance with completion.

The techniques employed for assessing non-motor outcomes in our study build upon the methodology outlined in our previous publication that investigated outcomes 30 days post-stroke, utilising PROMIS-29, and the Barthel Index.^18^ In addition to these established measures, our current study incorporates the SIS-59 v3.0, which shares a close relationship with PROMIS-29, but is designed to be stroke-specific; it comprehensively assesses activities of daily living (ADL), instrumental activities of daily living (IADL), communication, emotion, memory, thinking, social relationships, and features an additional visual analogue question on stroke recovery, rated on a scale from 0 to 100. Each item on the SIS-59 is evaluated using a 5-point Likert scale, and domain scores range from 0 to 100, calculated as follows: Domain score = [(Mean item score–1)/5–1] x 100.^19^

In our analysis, we have standardised each non-motor outcome scale score so that higher scores indicate worse outcome; we then dichotomised each non-motor domain as present or absent. Briefly, we considered a non-motor domain present if the item score was ≥55 in PROMIS-29, >2 in Barthel index scale, and ≥50 in SIS-59. To address our focus on non-motor outcomes we excluded the physical function domain of PROMIS-29, as well as strength, hand function, and mobility of SIS-59. For the same reason, we excluded the feeding, bathing, grooming, dressing, toilet use, transfers, mobility, and stairs domains of the Barthel Index.

All outcome measures used in our study are widely validated for proxy use. Therefore, we made every effort to include all eligible patients by implementing supplementary supportive measures for follow-up. For those patients with moderate to severe communication problems, severe cognitive impairment, language barriers such as none-English speaking, or severe functional disability (mRS 4–5) who could not travel to outpatient clinics, options included extended time, telephone follow-up, postal questionnaires, or translated outcome measures with a proxy responder's assistance.

### Standard protocol approvals, registrations, and patient consent

The SIGNAL registry was approved by the UCL Hospitals NHS Foundation Trust Governance Review Board (code: 5-201920-SE). Since the study data were collected as part of routine clinical care, the requirement for individual patient consent was waived.

### Statistical analysis

The statistical analysis plan for this project was developed jointly by H.O. and G.A. on December 18, 2019, and was in place prior to the completion of data collection. Patient demographics, clinical characteristics, and non-motor outcome domain scores were summarised with descriptive statistics. The continuous variables were analysed as means (Standard deviations, SD), medians (inter quartile range, IQR), and categorical variables were as numbers (n) and percentages (%). We used histograms and q–q plots to understand the distribution of the data. Unadjusted prevalence estimates were provided for 13 non-motor outcome domains as proportions with their 95% confidence intervals (Cl).

We performed logistic regression models to examine the univariable and multivariable associations between baseline sociodemographic and clinical factors with each non-motor outcome domain. The model-building process involved including any variables with univariate associations with p value < 0.20 were into a saturated multivariable model. Stepwise backward selection was used to remove variables individually until all remaining confounding variables were statistically significant at p value < 0.10, as well as clinically relevant baseline variables including age, sex, ethnic origin, stroke type, and stroke severity. Results from the adjusted multivariable logistic regression analysis were reported as odd ratios (OR) with their 95% Cl and p values. Additionally, correlation analyses were performed in which we calculated *Kappa* statistics to quantify the co-occurrence of non-motor outcome pairs.

All analysis were performed using STATA/MP version 18, and statistical significance was set at p < 0.05.

### Patient consent for publication

Not required.

### Role of the funding source

The funding sources had no role in data collection, analysis, interpretation, writing of the manuscript, or the decision to submit for publication.

## Results

### Patient characteristics

4107 acute stroke patients were admitted to the UCLH Comprehensive Stroke Service between 3rd January 2017 and 13th January 2020. Of these, 127 did not meet the eligibility criteria, and 642 patients deceased before follow-up (see Supplementary Figure S1). Therefore, 3338 patients who met our inclusion criteria were alive at 6-months post-stroke. Non-motor outcome data were available for 3080/3338 (92.3%) of eligible patients. Of these 127 (4.1%) patients received help from a proxy responder.

The baseline sociodemographic and clinical characteristics are summarised in Table 1. Briefly, 82.2% of participants had ischaemic stroke and 17.8% had ICH. The median age at stroke onset was 71.2 years (IQR 62–83), 1379 (44.8%) were female, 1774 (59%) were from white ethnic origin, 1024 (33.2%) Asian or Black ethic origin, median NIHSS score on hyperacute stroke unit (HASU) admission was 6 (IQR 5–12), mRS score at hospital discharge 3 (1–5) and 537 (18.1%) had a history of previous stroke/TIA. The median time from acute stroke onset to follow-up was 194.5 days (interquartile range, IQR 129.3–208).

**Table 1:** Baseline patient characteristics.

	All patients (N = 3080)
Age, mean (SD) y	71.2 ± 14.6
Female sex, N (%)	1379 (44.8%)
**Stroke type, N (%)**	
Ischaemic stroke	2534 (82.2%)
ICH	547 (17.8%)
**Ethnicity, N (%) (2994)**	
White	1774 (59.3%)
Asian	505 (16.9%)
Black	519 (17.3%)
Other	196 (6.6%)
**Medical history, N (%)**	
Previous stroke/TIA	537/2967 (18.1%)
Hypertension	1966 (63.8%)
Dementia	98 (3.2%)
Congestive heart failure	345/2964 (11.6%)
Diabetes Miletus	672/2956 (22.7%)
AF	611/3044 (20.1%)
Smoking history	803/2973 (27.0%)
**Medication at hospital admission, N (%)**	
Antiplatelet	1953/3006 (65%)
Anticoagulant	534/3001 (17.8%)
Antihypertensive	1853/3017 (61.4%)
Statin	1820/3006 (60.5%)
**Clinical outcomes (median, IQR)**	
Admission NIHSS (2993)	6 (5–12)
Pre-Morbid mRS (3074)	0 (0–1)
Discharge mRS (3019)	3 (1–4)
6 Months mRS	1 (0–3)
**Discharge location, N (%)**	
Home with ESD	1897/2903 (65.4%)
ASU	799/2903 (27.5%)
Care home	207/2903 (7.1%)
Proxy responders	127 (4.1%)
Length of HASU stay (days; median, IQR)	3 (1–5)
Time to follow-up, months	194.5 days ±2 weeks

ICH, Intracerebral haemorrhage; TIA, Transient ischaemic attack; AF, Arterial fibrillation; NIHSS, National Institute of Health Stroke Scale; mRS, modified Rankin Scale; ESD, Early supported discharge; ASU, Acute stroke unit; HASU, Hyperacute stroke unit.

The sociodemographic and the clinical data were analysed using descriptive statistics, continuous variables as means (Standard deviations, SD), medians (inter quartile range, IQR), and categorical variables as numbers (n) and percentages (%).

### Non-motor outcomes

**Fig. 1:**
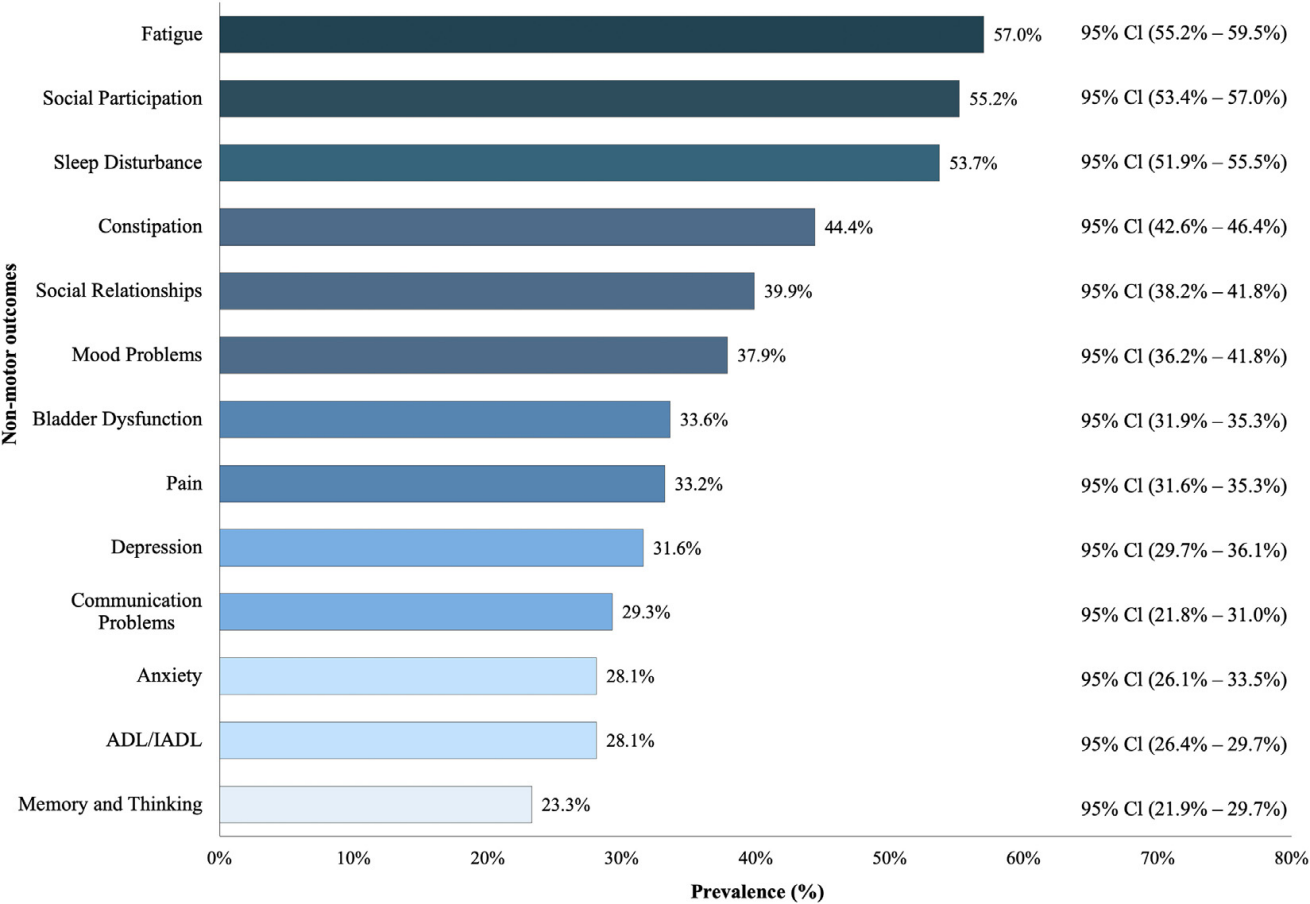
*Prevalence of adverse non-motor outcomes*. Prevalence estimates were calculated using unadjusted proportion analysis with their 95% Confidence Intervals.

The 6-month prevalence estimates of all 13 non-motor outcomes are shown in Fig. 1. Briefly, the most prevalent adverse non-motor outcomes were: fatigue N = 1756 (57%); reduced social participation N = 1694 (55%); sleep disturbance N = 1663 (54%); and constipation N = 1355 (44%). Less common adverse non-motor outcomes were: memory and thinking N = 708 (23%); ADL/IADL N = 860 (28%); anxiety N = 860 (28%); and communication problems N = 892 (29%).

**Fig. 2:**
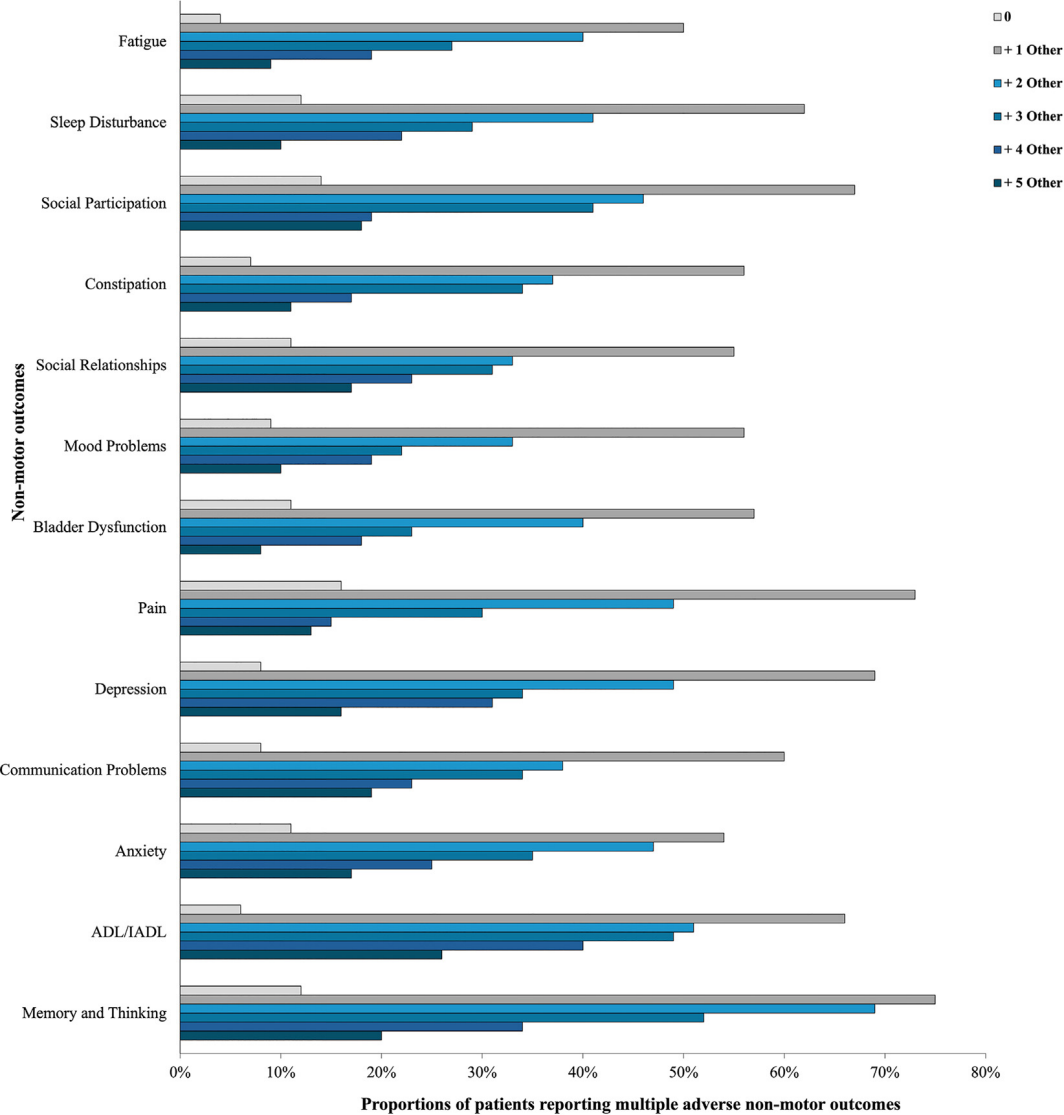
*Co-occurrence of adverse non-motor outcomes*. Bars show the proportions of patients with each adverse non-motor outcome who have additional adverse non-motor outcomes in ≥1, ≥2, ≥3 ≥ 4 and ≥5 other domains.

In our multi-domain analysis (see Fig. 2), N = 2310 (75%) of patients reported more than one adverse outcome, N = 1519 (49%) reported three, and N = 801 (26%) reported more than five adverse non-motor outcomes. All non-motor domains were most frequently reported in association with at least one additional adverse outcome rather than in isolation. Patients most frequently reporting one or more adverse non-motor outcomes included those experiencing problems with memory and thinking, pain, depression, and reduced social participation.

**Fig. 3:**
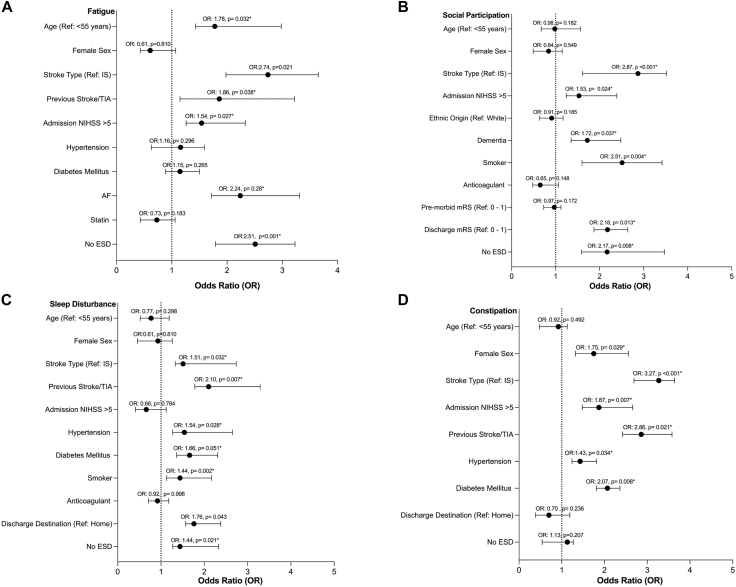
*Patient characteristics associated with adverse non-motor outcomes*. Each figure shows the results of multivariable analysis for individual non-motor domains across multiple outcomes, detailing the associated sociodemographic and clinical factors. Adjusted odds ratios (aORs) with 95% confidence intervals (CIs) are presented, where ORs greater than 1 indicate a significant positive association, while ORs equal to or less than 1 indicate no significant association. Each outcome domain is analysed separately, highlighting the diverse influences of sociodemographic and clinical variables on health outcomes. Logistic regression model of non-motor outcome domains, ranked by prevalence: A) Fatigue, B) Reduced Social Participation, C) Sleep Disturbance, and D) Constipation. The model is adjusted for clinical and sociodemographic characteristics (i.e., age, sex, stroke type, stroke severity, and cardiovascular risk factors).

The results of the adjusted multivariable analysis of baseline predictors of the most common 4 adverse non-motor outcomes are shown in Fig. 3; the predictors of other adverse non-motor outcomes are shown in Supplementary Figure S6. The factors most strongly associated with adverse non-motor outcomes were stroke due to ICH, admission NIHSS score>5, previous history of stroke or TIA, and a history of cardiovascular disease. Regarding predictors of the most common individual non-motor domains, fatigue was significantly associated with ICH (aOR 2.74, 95% CI 1.98–3.65, p = 0.021), admission NIHSS score >5 (aOR 1.54, 95% CI 1.26–2.33, p = 0.027), previous history of stroke/TIA (aOR 1.86, 95% CI 1.15–3.22, p = 0.038), and atrial fibrillation (aOR 2.24, 95% CI 1.72–3.31, p = 0.028). For reduced social participation, significant associations were found with ICH (aOR 2.87, 95% CI 1.61–3.52, p < 0.001) and admission NIHSS >5 (aOR 1.53, 95% CI 1.24–2.39, p = 0.024), while no significant associations were identified with previous history of stroke/TIA or cardiovascular risk factors. For sleep disturbance, significant associations were observed with ICH (aOR 1.51, 95% CI 1.33–2.74, p = 0.032), previous history of stroke/TIA (aOR 2.10, 95% CI 1.78–3.29, p = 0.007), and hypertension (aOR 1.54, 95% CI 1.27–2.65, p = 0.028), with no significant association found with admission NIHSS score. For constipation, significant associations were found with ICH (aOR 3.27, 95% CI 2.69–3.64, p < 0.001), previous history of stroke/TIA (aOR 2.86, 95% CI 2.42–3.58, p = 0.021), admission NIHSS >5 (aOR 1.87, 95% CI 1.48–2.66, p = 0.007), and hypertension (aOR 1.43, 95% CI 1.24–1.82, p = 0.034).

**Fig. 4:**
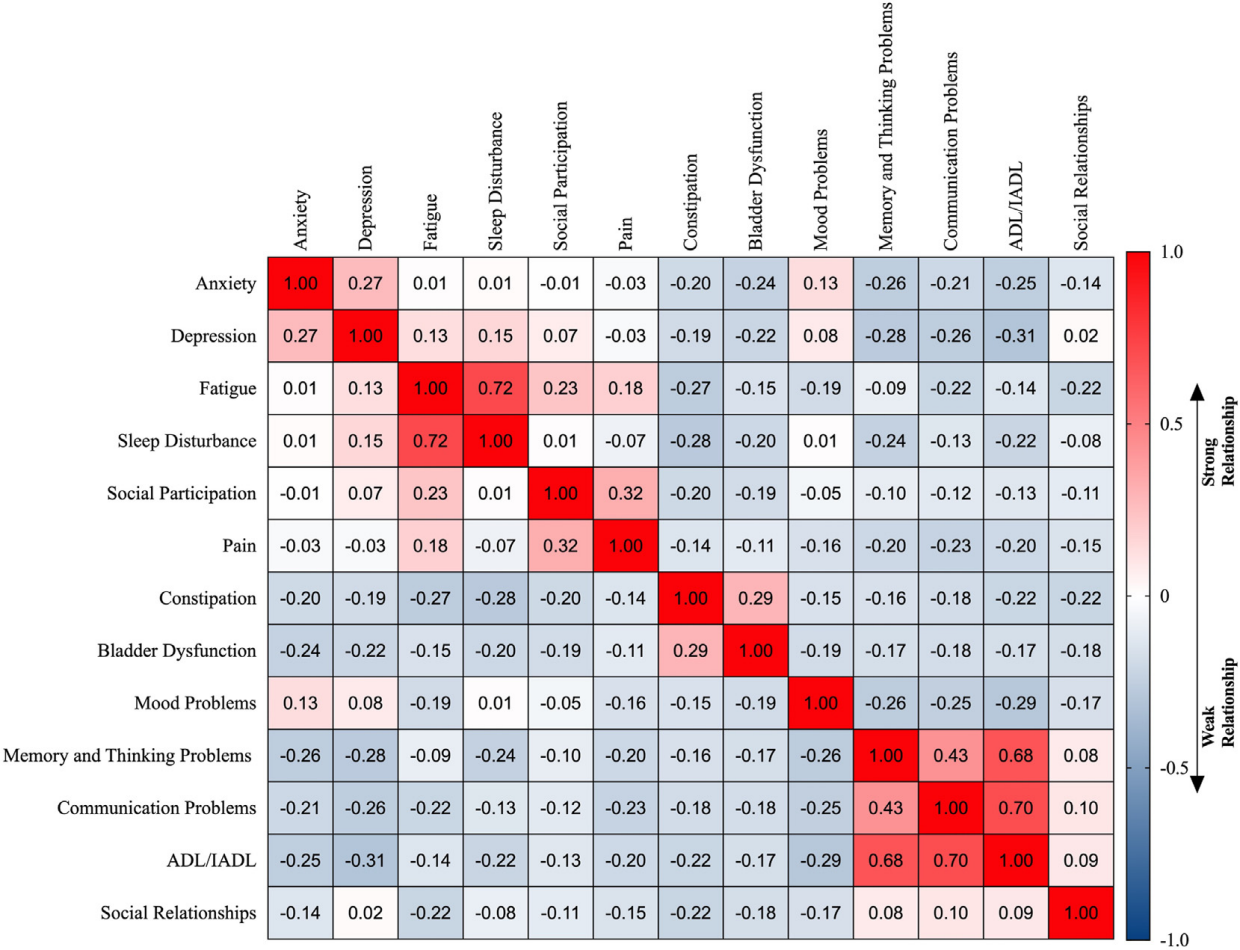
*Correlations between adverse non-motor outcomes*. Heatmap illustrating the correlations between individual adverse non-motor outcome domains. The intensity of the colour reflects the strength of the correlation, with red indicating a strong positive correlation and blue indicating a weak or no correlation.

The results of between-domain correlation analyses are shown in Fig. 4. We identified substantial correlation between fatigue and sleep disturbance (k = 0.72), reduced memory and thinking impairment and reduced ADL/IADL (k = 0.68), and between communication problems and ADL/IADL (k = 0.70), but no other statistically significant correlations between adverse non-motor outcomes.

## Discussion

Our large, ethnically diverse large prospective cohort study provides new comprehensive data on the prevalence, predictors, and patterns of adverse patient-reported non-motor outcomes in 13 health domains at 6 months after stroke. Our major finding is that adverse non-motor outcomes are highly prevalent and often multiple: 75% of stroke survivors have at least one affected domain; fatigue, sleep disturbance, and reduced social participation each affect over 50% of survivors; and rates of adverse non-motor outcomes in ⩾1, ⩾2, ⩾3, ⩾4, and ⩾5 domains were 75%, 51%, 49%, 40%, and 26%, respectively. In those with the highest burden of adverse non-motor outcomes (i.e., ≥5), memory and thinking problems, pain, depression, and reduced social participation were common. These observations indicate a previously unsuspected degree of complexity and co-occurrence in these non-motor outcomes, highlighting an urgent need to better detect and mitigate them to improve quality of life after stroke. Our correlation analysis revealed moderate associations among only three pairs of non-motor outcomes (fatigue and sleep disturbance; reduced memory and thinking impairment and reduced ADL/IAD; communication problems and ADL/IADL), but no other significant correlations; these findings suggests that each of the non-motor health domains requires independent assessment in clinical practice. Finally, we have identified baseline factors associated with adverse non-motor outcomes at 6 months, including stroke due to ICH, stroke severity, previous history of stroke/TIA, and a history of cardiovascular disease; these factors could help to identify individuals at highest risk, in whom specific interventions could be targeted to prevent or mitigate adverse non-motor outcomes.

Previous studies of non-motor outcomes after stroke are largely limited to investigating one or two non-domains, and very few have explored inter-domain correlations. Nevertheless, in line with our findings previous studies of individual domains estimated the prevalence of fatigue to be from 23% to 77%, sleep disturbance 17%–55%, and constipation 33%–63%, but no previous estimates were available to compare our results for reduced social participation.^19^, ^20^, ^21^, ^22^, ^23^ The variability in previous estimates are likely due to the methodological differences, such as time to follow-up, assessment measure, cohort size, and stroke type.^19^, ^20^, ^21^, ^22^, ^23^, ^24^ In previous studies, it was reported that fatigue is multidimensional, and is correlated with other non-motor outcomes such as sleep disturbance, pain, depression, anxiety, and memory problems.^20^ Our results also show moderate correlation between fatigue and sleep disturbance, but in contrast to the previous studies, we did not find significant correlations between fatigue and other non-motor outcomes. Nevertheless, anxiety, depression, memory and thinking problems co-occurred with fatigue but not pain. We did not find previous data to compare with our findings for reduced social participation and constipation, but for sleep disturbance a small cohort study (N = 112) reported that sleep quality independently accounts for fatigue at 6 years after stroke.^25^ While our study found no strong correlation between sleep disturbances and memory or thinking problems, previous research in non-stroke populations indicates a negative influence of sleep problems with speed of cognition and concentration.^25^^,^^26^ Since we only assessed self-reported subjective memory, further studies in stroke cohorts are needed to investigate the relationship between sleep and other cognitive domains.

We are aware of only one large-scale previous study (N = 2181)^27^ which investigated 7 non-motor outcome domains across various stroke types including ischaemic stroke, intracerebral haemorrhage, subarachnoid haemorrhage and transient ischaemic sttack.^27^ In addition to the inclusion of this heterogeneous cohort, other limitations included data collection in clinic (i.e., a convenience sample) rather than at a defined follow-up time after stroke and the collection of 25% of the data from proxy responders.^27^ Nevertheless, consistent with our results, the previous study highlighted that most affected domains were reduced social participation, and fatigue. However, in contrast to our findings, the study did not observe a high burden of sleep disturbance, or constipation^27^ which might be because of cohort heterogeneity and a focus on mean scores rather than dichotomised prevalence estimates.^27^ The previous study showed an independent association between non-motor outcomes post-stroke and functional disability measured by mRS and ICH.^27^ Our results showed a significant association between ICH with multiple adverse non-motor outcomes, but functional disability measured by the mRS was only associated with reduced social participation and pain, but not other non-motor outcomes.

We report comprehensive large-scale data indicating that patients with ICH, in comparison to those with ischaemic stroke, experience a higher risk of adverse non-motor outcomes in multiple domains, including anxiety, fatigue, sleep disturbance, reduced social participation, constipation, bladder dysfunction, mood problems, and social relationships. Moreover, this finding did not appear to be due strokes due to ICH being more severe, since the results were similar when adjusted for stroke severity and discharge mRS. Our findings align with a large cohort study (N = 1064) indicating that ICH in stroke patients correlates with poorer mental health.^12^ Moreover, One small study including 377 participants (307 with cerebral infarction, 44 with TIA and 26 with intracerebral haemorrhage) at a mean follow-up time of 382 days found that fatigue was more common after ischaemic stroke than ICH (45.9% vs. 19.2%) but had limited power to adjust for stroke severity.^28^^,^^29^ Our previous study (n = 605) found that patients with ICH had higher rates of reduced social participation, sleep disturbance, bladder dysfunction, and mood problems at 30-days^18^; our current study confirms that these common adverse non-motor outcomes in patients with ICH persist at 6 months. Regarding constipation, a meta-analysis study involving 8 small cohort studies suggest that patients with ICH were more likely to report constipation than ischaemic stroke (66% vs. 51%),^24^ in line with our findings, though our large sample size allowed us to adjust for other factors suggesting that ICH is an independent risk factor for this adverse outcome. The biological explanations for why ICH is independently associated with adverse non-motor outcomes, even after adjusting for confounding factors including stroke severity, remain uncertain. The mechanisms of brain injury after ICH are different to those following ischaemic stroke; these are complex and include direct tissue injury, damage from toxic blood breakdown products and extravasated plasma proteins, as well as perihematomal oedema and both local and generalised brain inflammation. Other relevant mechanisms include subarachnoid extension, which occurs in CAA, and intraventricular ICH with hydrocephalus, which may have contributed to the worse outcomes.^24^^,^^28^^,^^29^ How and whether these mechanisms might relate to poorer non-motor outcomes after ICH require further investigation, ideally using both neuroimaging and fluid biomarkers.

Another finding from our study is the association between a history of previous stroke and multiple adverse non-motor outcomes. Data on the consequences of recurrent vs first-ever stroke are limited, but one study in 1138 unselected patients with acute stroke found an association between recurrent ischaemic stroke with greater functional dependence and increased mortality.^30^ Furthermore, a post-hoc analysis of a previous study on TIA and minor ischaemic stroke indicated that stroke recurrence at 3-months was associated with poor non-motor outcomes.^31^ Although these findings align with our results the previous study was limited to individuals with better neurological function, only investigated 5 non-motor domains, and did not provide data from patients with ICH.^30^^,^^31^

We also found that atrial fibrillation is associated with adverse prevalence of depression, fatigue, mood problems, and reduced ADL after stroke, in line with a previous study evaluating patient-reported health after stroke.^12^

The prognostic value of the NIHSS score is well-documented, with numerous studies associating higher admission scores with long-term adverse motor and non-motor outcomes.^13^^,^^32^ As expected, patients with higher NIHSS scores typically face greater challenges, including prolonged rehabilitation and a higher likelihood of residual disabilities.^13^^,^^32^, ^33^, ^34^, ^35^, ^36^ In line with several previous studies, our adjusted results for the association between NIHSS score and multiple adverse non-motor outcomes confirm and extend previous findings^13^^,^^32^, ^33^, ^34^, ^35^, ^36^, ^37^, ^38^, ^39^; previous data on people with ICH have been limited and most often examining associations of NIHSS with only 2–5 non-motor outcome domains.^13^^,^^24^, ^25^, ^26^, ^27^, ^28^, ^29^^,^^30^^,^^31^

Our study has important strengths. We included a large, unselected cohort from an unselected ethnically diverse population, reducing the likelihood that the results are biased towards a specific ethnicity. Moreover, we achieved a very high rate of follow-up by using multiple methods including administration of questionnaires in a local outpatient stroke clinic, telephone follow-up, face to face follow-up at individuals residential address, and help from proxy responders. This approach allowed efficient collection of detailed data on 13 non-motor health domains. The large sample size and complete baseline data also enabled us to adjust for important sociodemographic and clinical factors that impact adverse prevalence of non-motor outcomes. The inclusion of patients with both ischaemic stroke and intracerebral haemorrhage allowed us to explore differences across these stroke types.

Our study also has limitations. The non-motor data in our study is patient-reported therefore relies on individuals' perceptions and interpretations, which can over- or underestimate symptoms in some domains such as anxiety and depression. We also acknowledge that for some non-motor outcome domains PROMIS-29 measures symptom burden over the 7 days and does not provide a formal diagnosis of these conditions. Our data describe the burden of non-motor outcomes at 6 months, but more data are needed at longer-term follow-up. Although 1 in 2 individuals reports adverse non-motor outcomes across multiple domains, we do not have detailed data on patients’ pre-stroke non-motor outcome status. We used multiple follow-up methods (face to face, telephone, or postal) to maximise participation of patients, including those with severe disability, to minimise bias, but the different methods might have affected our results. Furthermore, we did not have data on any clinical interventions that may have been provided for most adverse non-motor outcomes (e.g., fatigue and sleep management, rehabilitation for social participation, support for anxiety, depression, and mood problems, management of communication problems or memory and thinking problems). Finally, future studies are needed to investigate the association between deprivation and the prevalence of non-motor outcomes.

In summary, our large observational study provides new evidence on the prevalence, predictors and patterns for adverse non-motor outcomes across 13 health domains, highlighting a high and complex burden on patients and carers. Patients with a history of ICH, prior stroke or TIA, high stroke severity and cardiovascular disease are at the highest risk. Our study contributes new and detailed data to inform the growing efforts from healthcare systems, stroke patient support organisations, and clinical guidelines to focus more on the lived experience of the long-term consequences of stroke, including adverse non-motor outcomes. Our data provide evidence to guide clinicians and policymakers in developing of effective clinical interventions for patient-dedicated stroke services.

## Contributors

HO helped shape the study by contributing to its design, overseeing the extraction of baseline data from electronic health records, participating in follow-up data collection, cleaning data, and conducting statistical analyses under the supervision of GA. JJM, CB, and SB were actively involved in collecting follow-up data and data entry. GB and AJL contributed to the study's design and offered valuable advice on the analysis process. RJS and DJW were involved in designing and supervising the entire study, providing consistent support and guidance throughout.

## Data sharing statement

Fully anonymised data included in this study will be shared upon request from the senior author pending appropriate Institutional Ethics/Governance Board approvals.

## Declaration of interests

DJW reports funding as an NIHR Senior Investigator, grant funding from the British Heart Foundation, and personal fees from Alnylam, AstraZeneca, Bayer, and NovoNordisk, outside the submitted work.
