# Supplementary material for: Prevalence, predictors, and patterns of patient reported non-motor outcomes six months after stroke: a prospective cohort study
Source: Lancet Reg Health Eur. 2024 Oct 19;47:101080. doi: 10.1016/j.lanepe.2024.101080 (PMC11532962; doi:10.1016/j.lanepe.2024.101080)
Supplement: Appendix [file mmc1.docx]

## Supplementary File: Prevalence, predictors, and patterns of patient reported non-motor outcomes 6 months after stroke: population-based prospective cohort study

| **Table of Contents** | **Page** |
| --- | --- |
| **Figure 1.** *Patient flowchart* | **2** |
| Figure 2. STROBE Statement | **4 – 6** |
| **Figure 3.** PROMIS-29 Scale | **7 – 9** |
| **Figure 4**. SIS-59 Scale | **10 – 14** |
| **Figure 5.** Barthel Index Scale | **15** |
| **Figure 6.** Adjusted Results E to M | **16 – 17** |
| **Table 1.** *Deceased patient characteristics* | **18** |

**Figure 1.** *Patient flowchart*

**
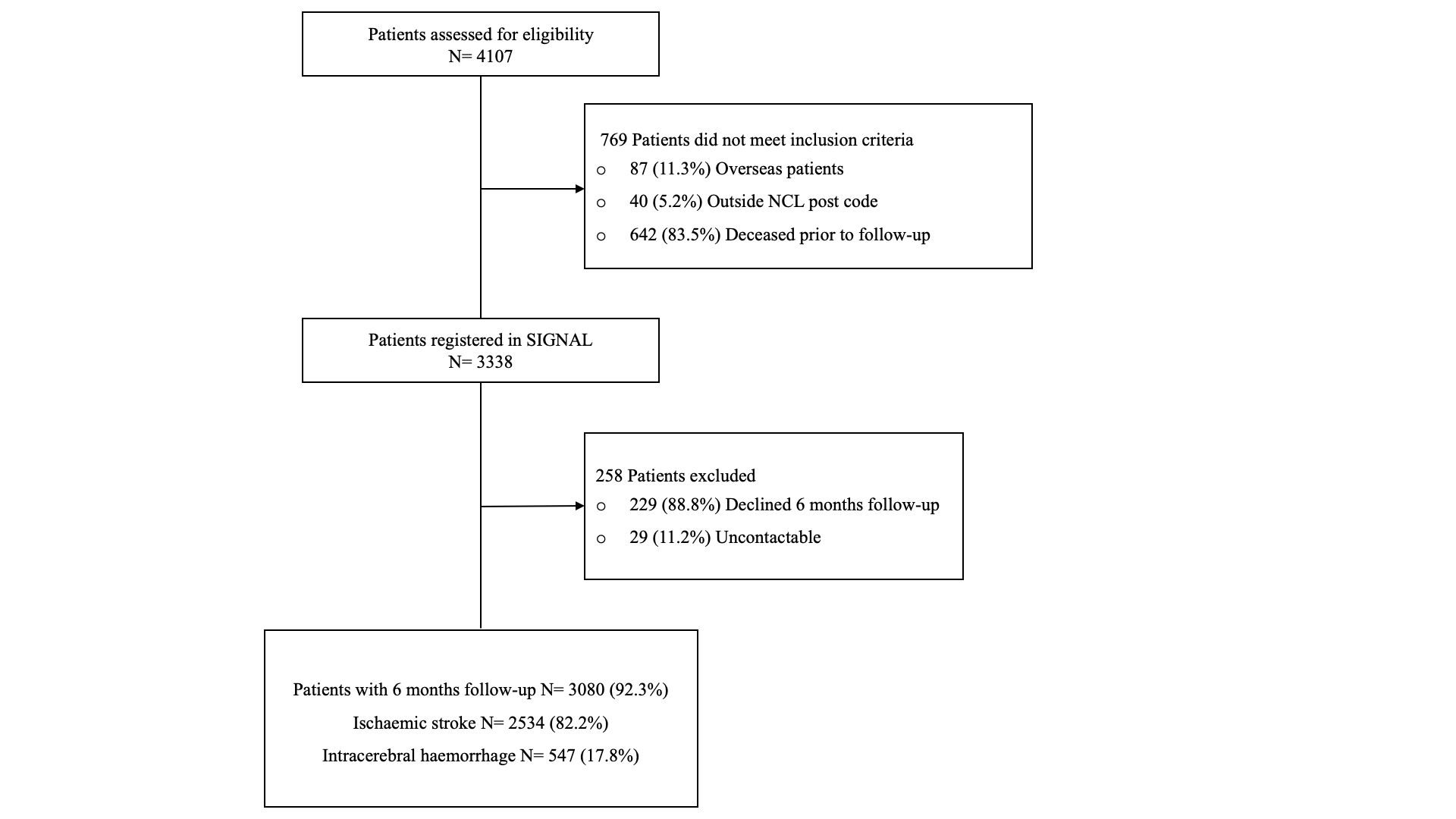
**

## Figure 2. STROBE Statement

|  | Item No. | Recommendation | Page  No. | |  | |
| --- | --- | --- | --- | --- | --- | --- |
| **Title and abstract** | 1 | (*a*) Indicate the study’s design with a commonly used term in the title or the abstract | 1 | |  | |
|  |  | (*b*) Provide in the abstract an informative and balanced summary of what was done and what was found | 2 | |  | |
| Introduction | | | | |  | |
| Background/rationale | 2 | Explain the scientific background and rationale for the investigation being reported | 5 | |  | |
| Objectives | 3 | State specific objectives, including any prespecified hypotheses | 5 | |  | |
| Methods | | | | |  | |
| Study design | 4 | Present key elements of study design early in the paper | 5, 8 | |  | |
| Setting | 5 | Describe the setting, locations, and relevant dates, including periods of recruitment, exposure, follow-up, and data collection | 5 | |  | |
| Participants | 6 | (*a*) *Cohort study*—Give the eligibility criteria, and the sources and methods of selection of participants. Describe methods of follow-up  *Case-control study*—Give the eligibility criteria, and the sources and methods of case ascertainment and control selection. Give the rationale for the choice of cases and controls  *Cross-sectional study*—Give the eligibility criteria, and the sources and methods of selection of participants | 5,6 (Figure 1 Supplementary File) | | |  |
|  |  | (*b*) *Cohort study*—For matched studies, give matching criteria and number of exposed and unexposed  *Case-control study*—For matched studies, give matching criteria and the number of controls per case |  |  | | |
| Variables | 7 | Clearly define all outcomes, exposures, predictors, potential confounders, and effect modifiers. Give diagnostic criteria, if applicable | 7,8 | |  | |
| Data sources/ measurement | 8* | For each variable of interest, give sources of data and details of methods of assessment (measurement). Describe comparability of assessment methods if there is more than one group | 7,8 | |  | |
| Bias | 9 | Describe any efforts to address potential sources of bias |  | |  | |
| Study size | 10 | Explain how the study size was arrived at 6 | (Figure 1 Supplementary File) | |  | |

Continued on next page

| Quantitative variables | 11 | Explain how quantitative variables were handled in the analyses. If applicable, describe which groupings were chosen and why | - |  |
| --- | --- | --- | --- | --- |
| Statistical methods | 12 | (*a*) Describe all statistical methods, including those used to control for confounding | 8 |  |
|  |  | (*b*) Describe any methods used to examine subgroups and interactions | 8 |  |
|  |  | (*c*) Explain how missing data were addressed |  |  |
|  |  | (*d*) *Cohort study*—If applicable, explain how loss to follow-up was addressed  *Case-control study*—If applicable, explain how matching of cases and controls was addressed  *Cross-sectional study*—If applicable, describe analytical methods taking account of sampling strategy | 8 |  |
|  |  | (*e*) Describe any sensitivity analyses |  |  |
| Results | | | | |
| Participants | 13* | (a) Report numbers of individuals at each stage of study—eg numbers potentially eligible, examined for eligibility, confirmed eligible, included in the study, completing follow-up, and analysed | 9,10 |  |
|  |  | (b) Give reasons for non-participation at each stage | 9 |  |
|  |  | (c) Consider use of a flow diagram | 9 |  |
| Descriptive data | 14* | (a) Give characteristics of study participants (eg demographic, clinical, social) and information on exposures and potential confounders | 9 |  |
|  |  | (b) Indicate number of participants with missing data for each variable of interest | 9 |  |
|  |  | (c) *Cohort study*—Summarise follow-up time (eg, average and total amount) | 9 |  |
| Outcome data | 15* | *Cohort study*—Report numbers of outcome events or summary measures over time | 9,10 |  |
|  |  | *Case-control study—*Report numbers in each exposure category, or summary measures of exposure | - |  |
|  |  | *Cross-sectional study—*Report numbers of outcome events or summary measures | 8,9  (Figure 2) |  |
| Main results | 16 | (*a*) Give unadjusted estimates and, if applicable, confounder-adjusted estimates and their precision (eg, 95% confidence interval). Make clear which confounders were adjusted for and why they were included | 9, 10  (Figure 3, page 21-22) |  |
|  |  | (*b*) Report category boundaries when continuous variables were categorized | - |  |
|  |  | (*c*) If relevant, consider translating estimates of relative risk into absolute risk for a meaningful time period |  |  |

| Other analyses | 17 | Report other analyses done—eg analyses of subgroups and interactions, and sensitivity analyses | 9,10 |  |
| --- | --- | --- | --- | --- |
| Discussion | | | | |
| Key results | 18 | Summarise key results with reference to study objectives | 10, 13 |  |
| Limitations | 19 | Discuss limitations of the study, taking into account sources of potential bias or imprecision. Discuss both direction and magnitude of any potential bias | 13 |  |
| Interpretation | 20 | Give a cautious overall interpretation of results considering objectives, limitations, multiplicity of analyses, results from similar studies, and other relevant evidence | 10-13 |  |
| Generalisability | 21 | Discuss the generalisability (external validity) of the study results | 10-13 |  |
| Other information | |  | | |
| Funding | 22 | Give the source of funding and the role of the funders for the present study and, if applicable, for the original study on which the present article is based | 2 |  |

**
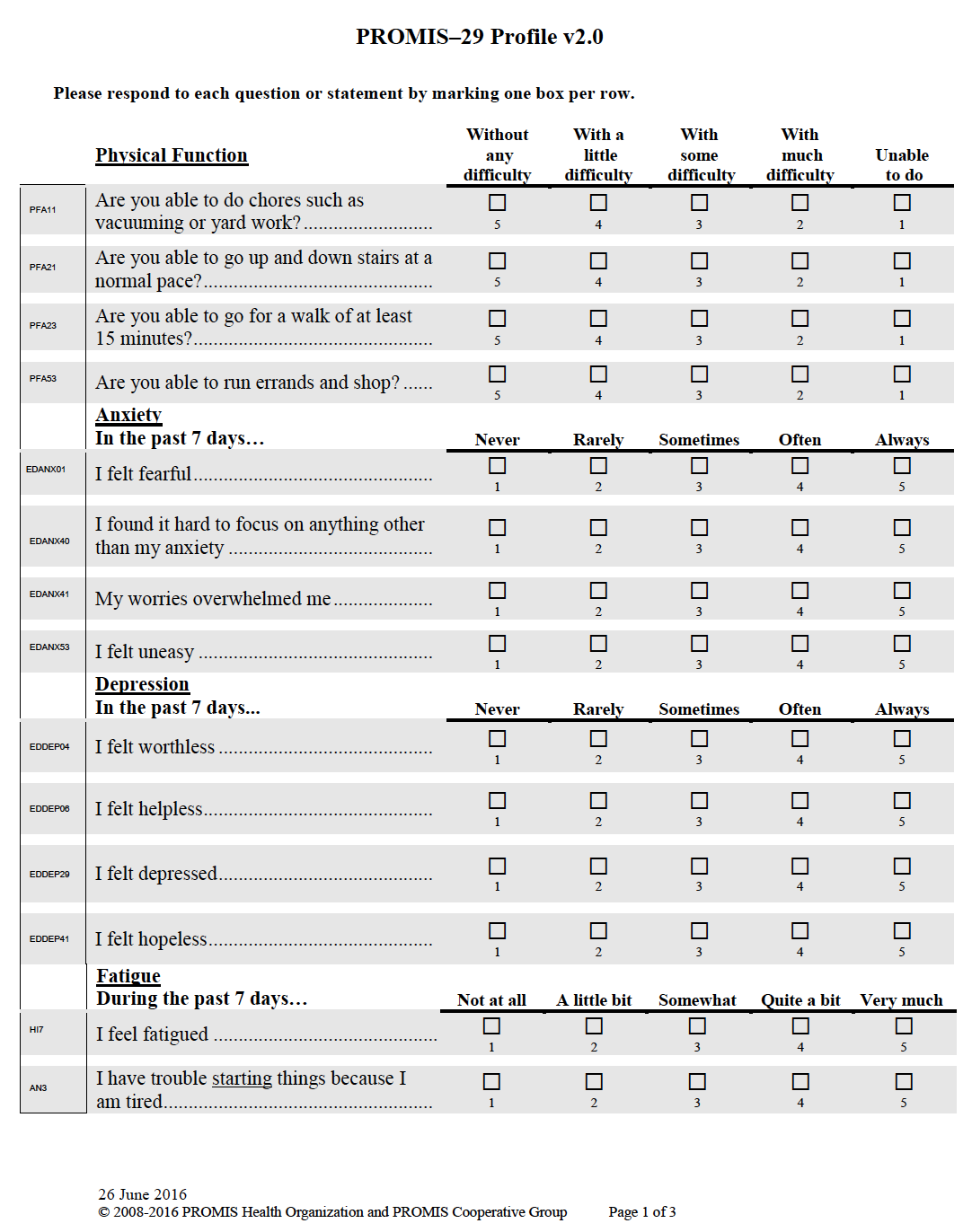
Figure 3.** PROMIS-29 Scale


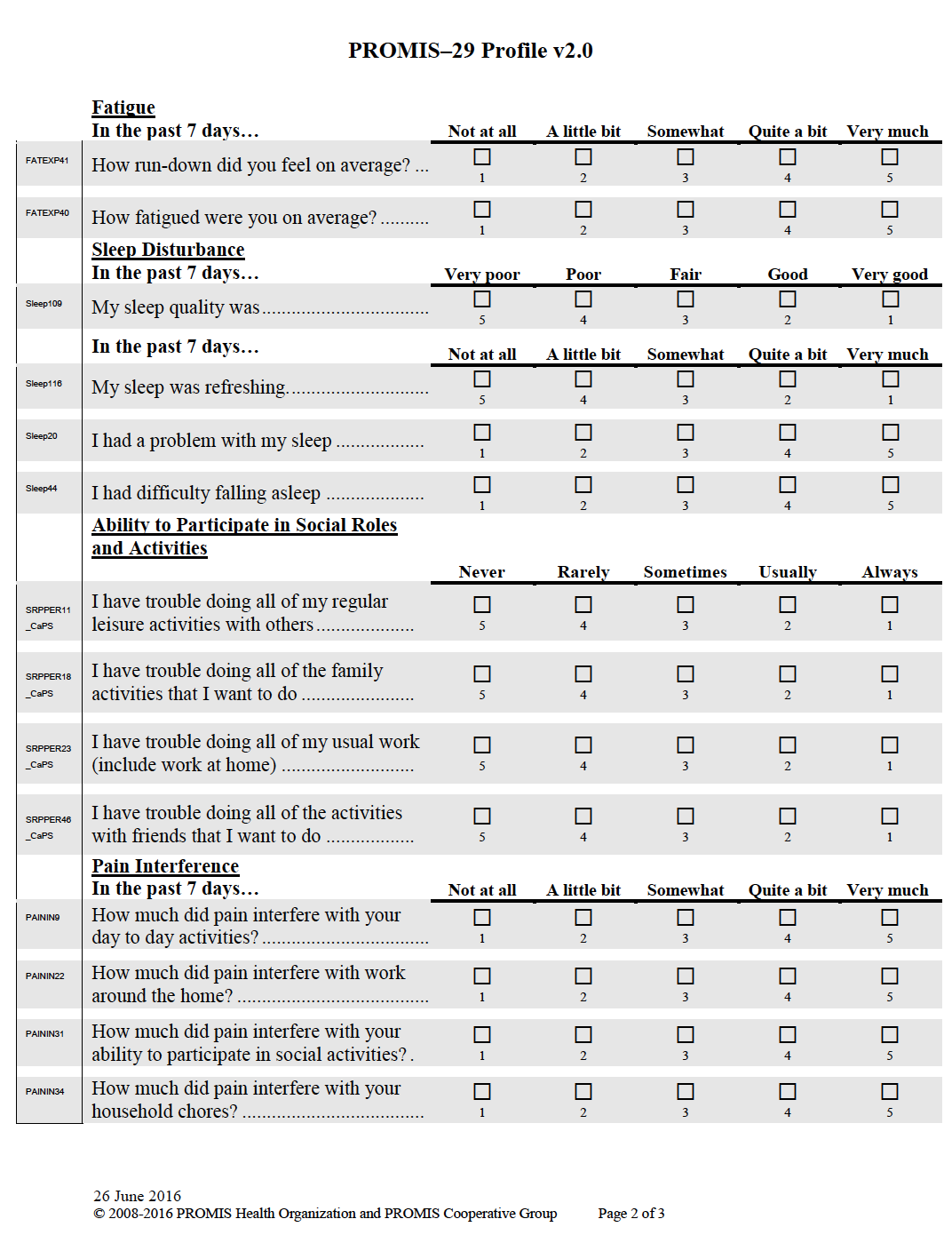


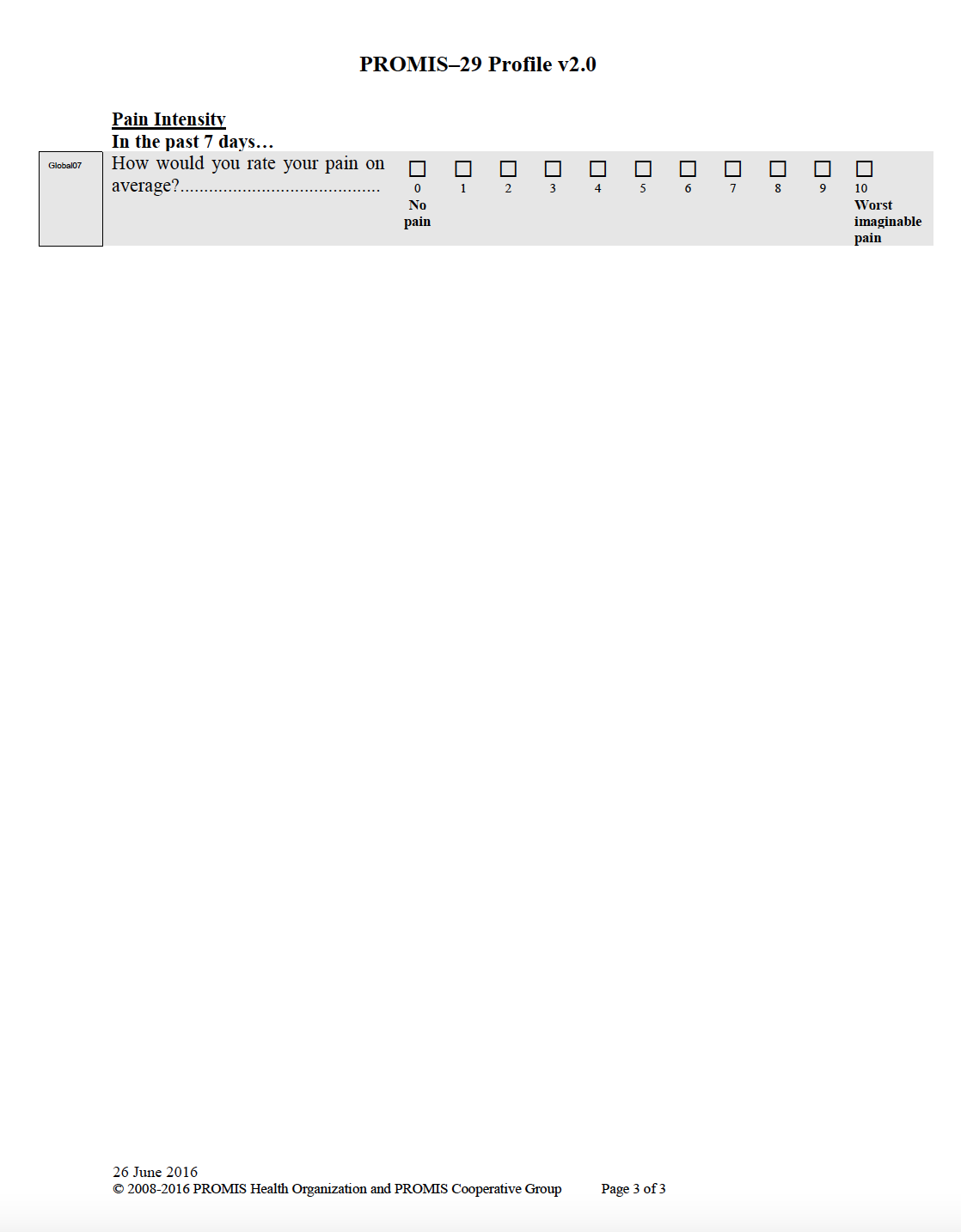


**Figure 4**. SIS-59 Scale


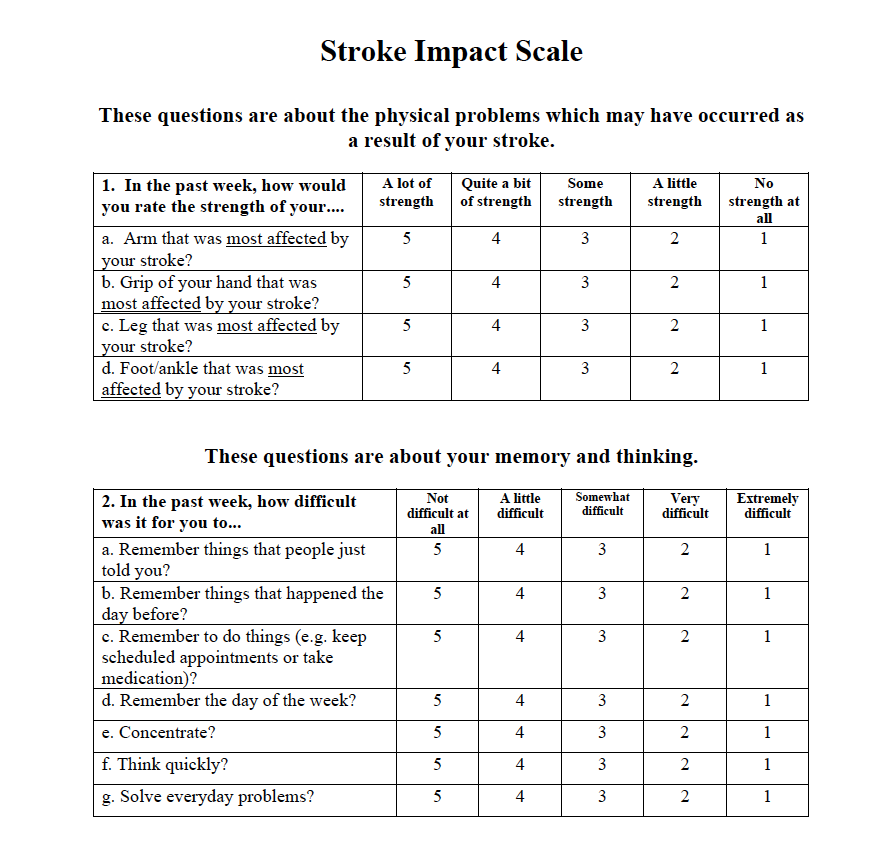


**
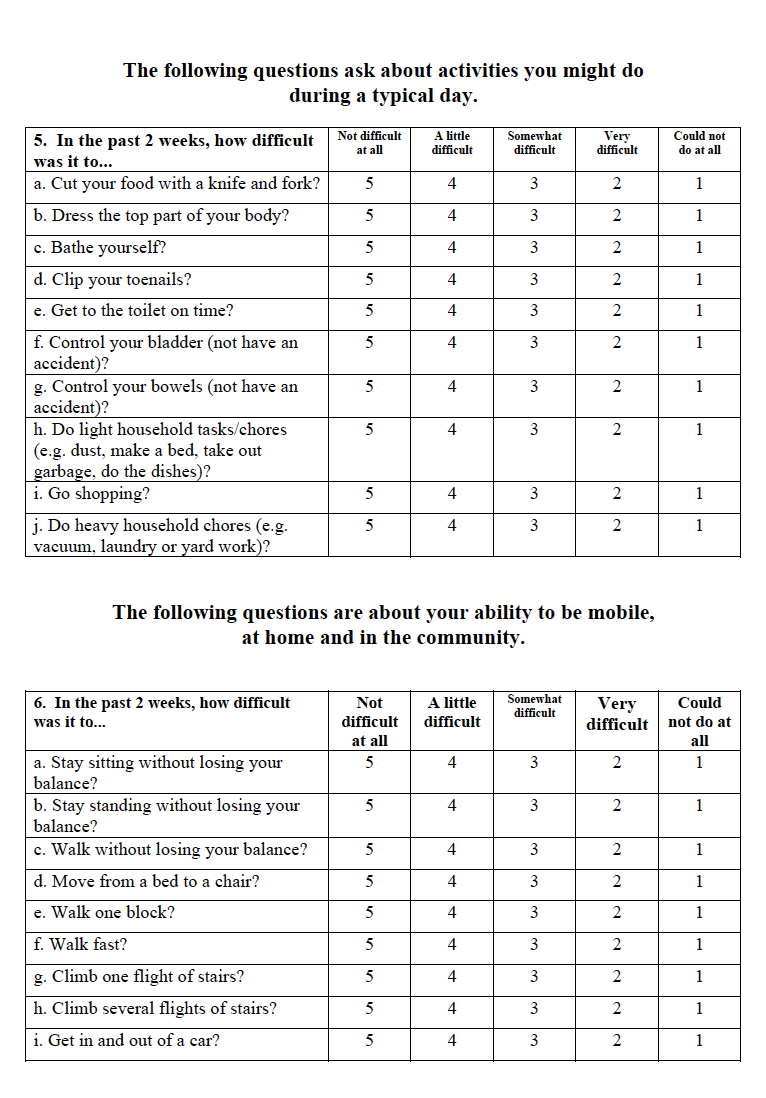
**

**
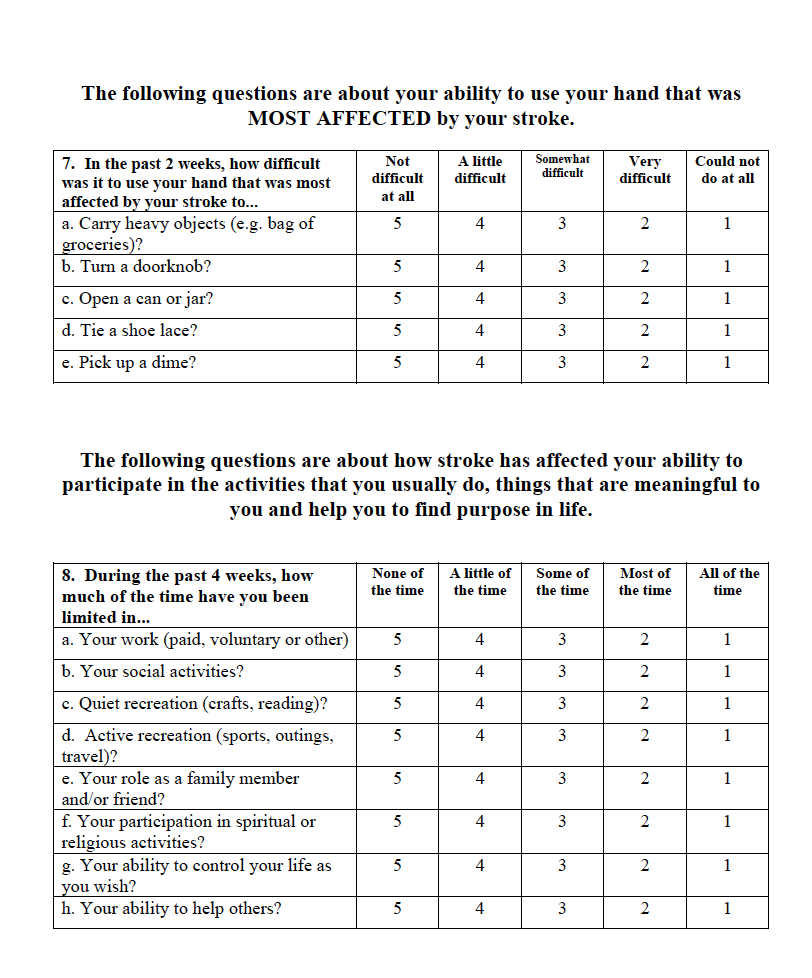
**


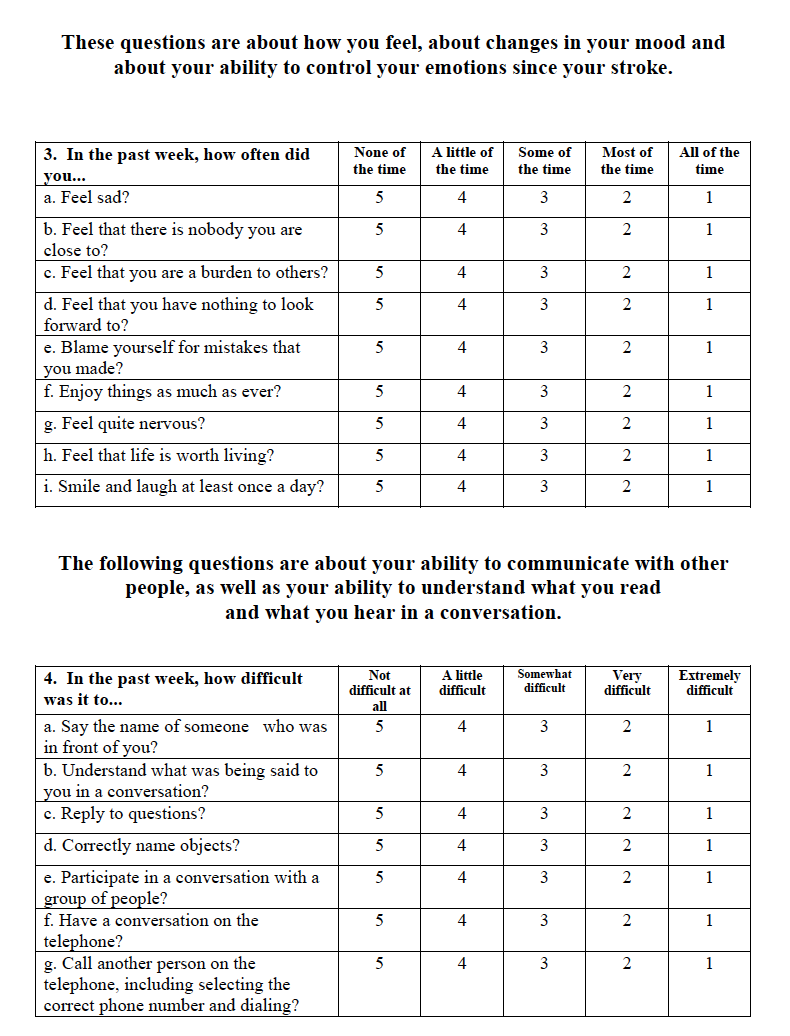


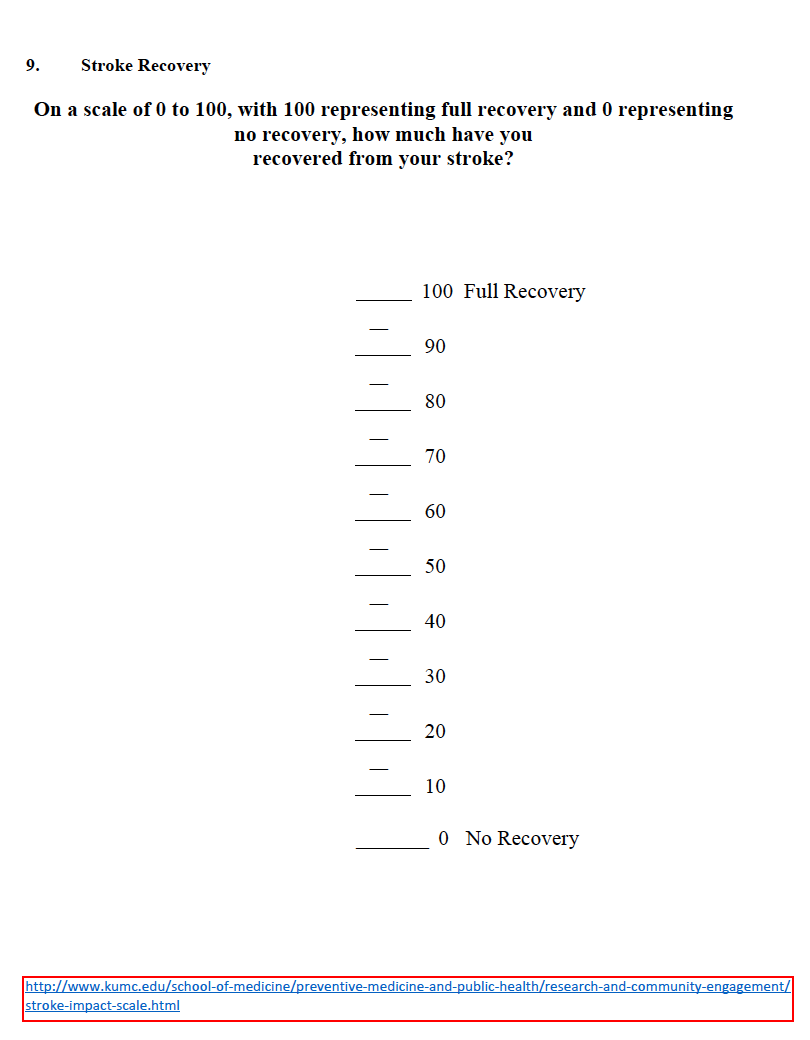


**
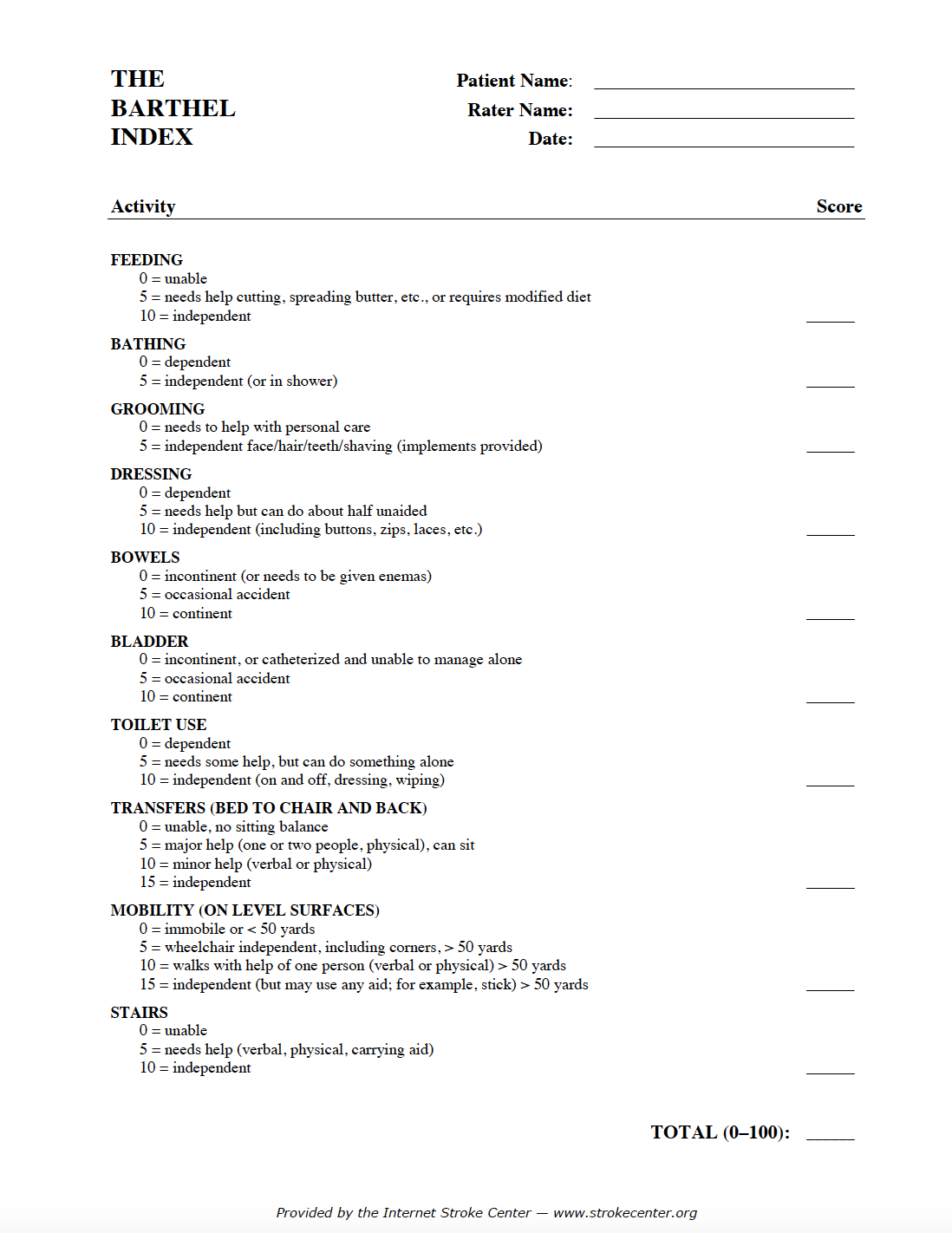
Figure 5.** Barthel Index Scale

**Figure 6.** *Adjusted predictors of non-motor outcomes domains (E-anxiety, F-depression, G-pain, H-bladder dysfunction, I-mood problems, J-memory problems, K-communication problems, L-social relationships and M-ADL)*


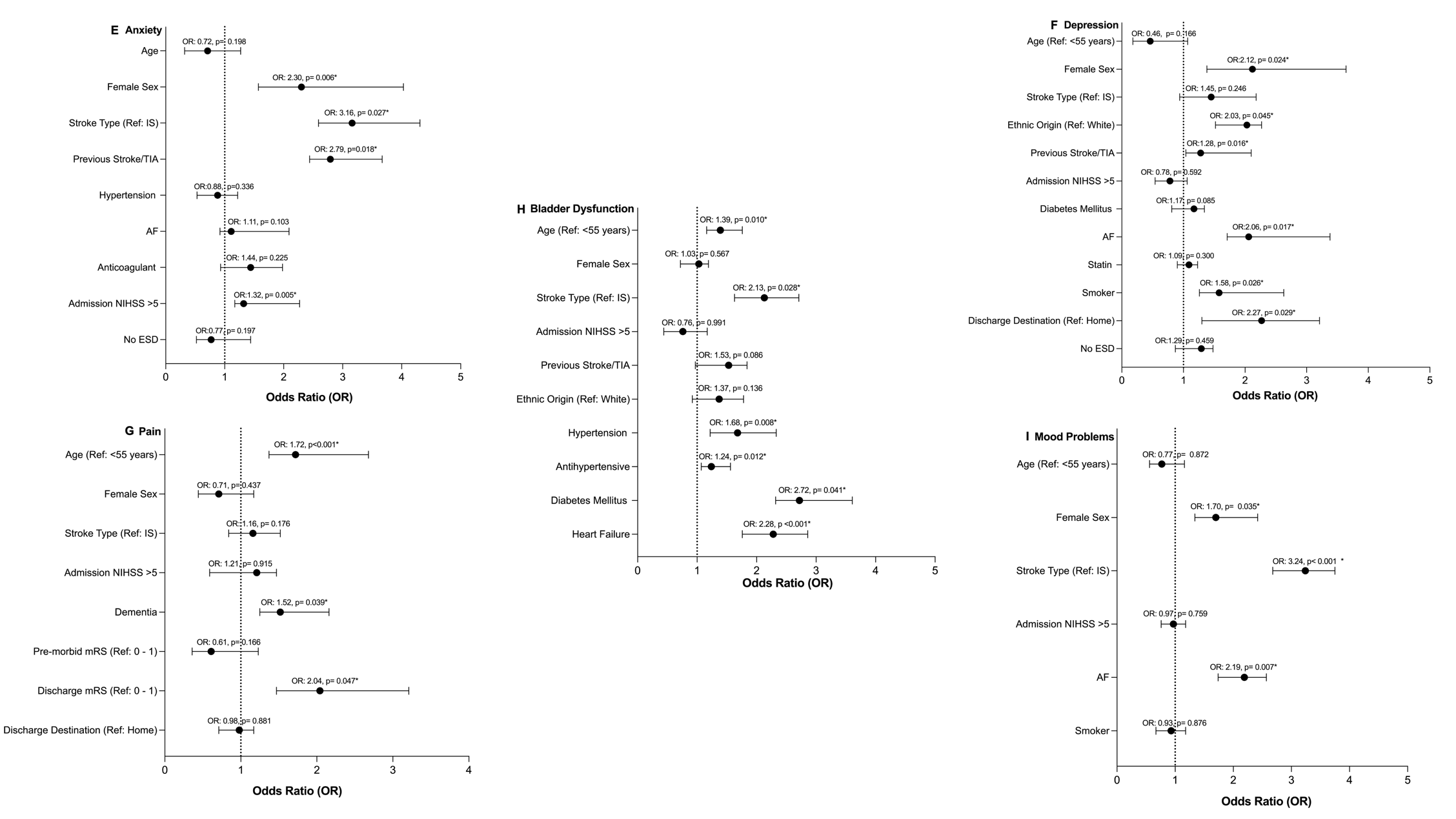


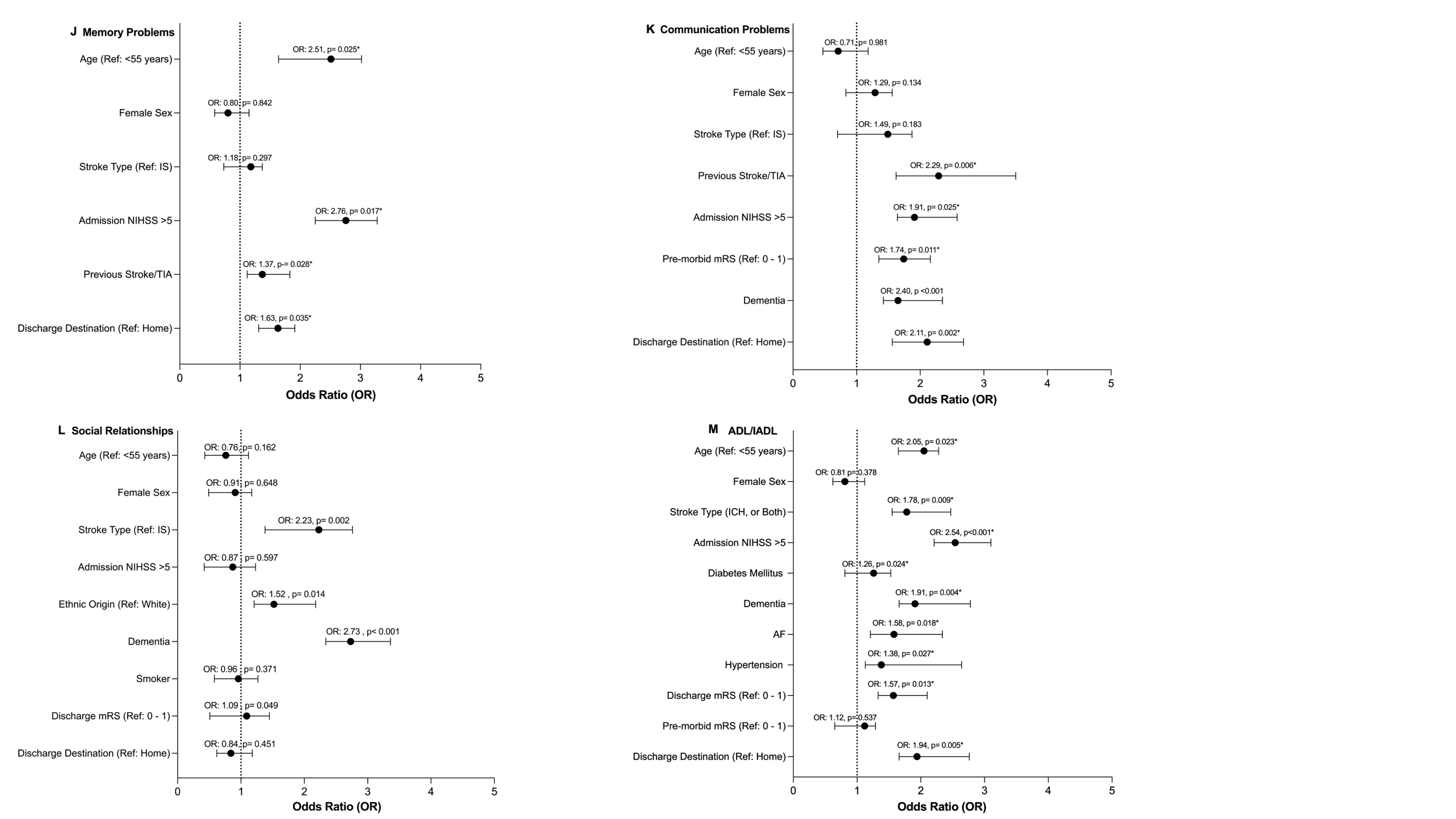


|  | Patients who deceased prior to follow-up  N= 642 |
| --- | --- |
| Age | 71 ± 13.2 |
| Female Sex | 279 (43.5%) |
| **Stroke Type** | |
| Ischaemic stroke | 510 (79%) |
| ICH | 132 (21%) |
| **Ethnicity, N (%) (629)** | |
| White | 493 (78%) |
| Asian | 38 (6%) |
| Black | 46 (7%) |
| Other | 57 (9%) |
| **Medical History, N (%)** | |
| Previous stroke/TIA | 206 (32%) |
| Hypertension | 461 (72%) |
| Dementia | 6 (0.9%) |
| Congestive Heart Failure | 41 (6.4%) |
| Diabetes Miletus | 156 (24.3%) |
| AF | 201 (31.3%) |
| Smoking History | 53 (8.3%) |
| **Clinical Outcomes (median IQR)** | |
| Admission NIHSS | 10 (4 – 18) |
| Pre-Morbid mRS | 3 (2 – 5) |

**Table 1.** *Characteristics of deceased patients*

*The sociodemographic and the clinical data were analysed using descriptive statistics, continuous variables as means (Standard deviations, SD), medians (inter quartile range, IQR), and categorical variables as numbers (n) and percentages (%). ICH= Intracerebral haemorrhage; TIA= Transient ischaemic attack; AF= Arterial fibrillation; NIHSS= National Institute of Health Stroke Scale; mRS= modified Rankin Scale.
